# Draft Genome Sequences of Marine RNA Viruses SF-1, SF-2, and SF-3 Recovered from San Francisco Wastewater

**DOI:** 10.1128/genomeA.00653-15

**Published:** 2015-06-18

**Authors:** Alexander L. Greninger, Joseph L. DeRisi

**Affiliations:** a Department of Biochemistry and Biophysics, UCSF, San Francisco, California, USA; b Howard Hughes Medical Institute, UCSF, San Francisco, California, USA

## Abstract

We report the draft genome sequences of marine RNA viruses SF-1, SF-2, and SF-3, which were assembled from metagenomic sequencing of organisms in San Francisco wastewater. These viruses were most closely related to marine RNA virus JP-B and algae viruses.

## GENOME ANNOUNCEMENT

The picorna-like superfamily is a rapidly expanding taxonomic unit of positive-stranded RNA viruses with conserved RNA-dependent RNA polymerase (RdRp), capsid, and helicase proteins that have a broad host range, including animals, plants, and insects (1–3). While performing weekly metagenomic sequencing of organisms in San Francisco wastewater, we assembled three contigs of 8,695, 9,270, and 8,642 nucleotides that aligned by BLASTx to the RNA-dependent RNA polymerase and capsid genes of the marine RNA virus JP-B (30 to 40% amino acid identity), a member of the *Picornavirales* order (1, 2). The first two contigs contained bicistronic viral genomes with open reading frames (ORFs) of 4,782/2,877 nucleotides (RNA-dependent RNA polymerase-containing polyprotein ORF/structural polyprotein ORF) and 5,178/2,664 nucleotides, while the third genome contained a single ORF of 7,731 nucleotides in the standard genetic code. The ORFs aligned by 30 to 38% to each other and ~30% by amino acids to RNA viruses of *Chaetoceros tenuissimus*, *Rhizosolenia setigera*, and *Asterionellopsis glacialis*, suggesting that these may be RNA viruses of algae.

All three contigs were assembled from a single metagenomic library derived from a wastewater sample taken on 25 January 2010 following a large rainstorm that left >5 inches of rain over the preceding week. Unlike the likely ciliate viruses also discovered in this sample, wastewater samples collected in March 2010 also contained reads to these viruses but at a significantly lower sequence count. This sample was created by 200-fold concentration of 1 liter of wastewater, with particles between the size of 0.22 μm and 300 kDa using Millipore Pellicon XL 300-kDa filters and 0.22-μm spin columns. The viral particle-enriched sample was treated with micrococcal nuclease, nucleic acid was extracted using the Zymo viral DNA/RNA kit, and half of the recovered nucleic acid was treated with DNase. The three contigs were discovered and assembled using PRICE version 1.0 (4), the Geneious version 8.0 Assembler, and SURPI version 1.0 (5) from a total of 15,719,690 paired-end 65-bp reads sequenced on an Illumina GAIIx split between these DNAsed and untreated nucleic acid preparations (3, 6). The average coverages of the three contigs using all reads from the sample were 1,749×, 1,379×, and 222×.

### Nucleotide sequence accession numbers.

The GenBank accession numbers for marine RNA viruses SF-1, SF-2, and SF-3 are JN661160, KF412901, and KF478836, respectively.
